# RNA-Seq Technology Reveals the Mechanism of SDT Combined With Novel Nanobubbles Against HCC

**DOI:** 10.3389/fmolb.2021.791331

**Published:** 2022-02-07

**Authors:** Haitao Shang, Yichi Chen, Chunyue Wang, Shentao Zhang, Bolin Wu, Xitian Liang, Zhao Liu, Qiucheng Wang, Wen Cheng

**Affiliations:** ^1^ Department of Ultrasound, Harbin Medical University Cancer Hospital, Harbin, China; ^2^ Department of Interventional Ultrasound, Harbin Medical University Cancer Hospital, Harbin, China

**Keywords:** nanobubble, hepatocellular carcinoma, sonodynamic therapy, RNA-seq, HMME, LND

## Abstract

Sonodynamic therapy is widely used in the treatment and research of hepatocellular carcinoma. A novel targeted nanobubble complex mediated with Hematoporphyrin monomethyl ether and Lonidamine was structured as a sensitizer, characterized the properties, and studied the therapeutic effect on hepatocellular carcinoma. The complexes can promote the apoptosis of hepatocellular carcinoma cells and work better in combination with sonodynamic therapy. The differential expression of multiple types of RNA in hepatocellular carcinoma with sonodynamic therapy can be identified accurately with high-throughput RNA sequencing. The differential expressions of mRNA, lncRNA, and circRNA were analyzed by RNA-Seq. The enrichment analyses (Gene Ontology and KEGG) prompted the meaningful genes and pathways in the process of sonodynamic therapy in hepatocellular carcinoma cells. HMME-LND@C_3_F_8_-NBs conjugated with ultrasound is confirmed efficiently for inhibiting the development of hepatocellular carcinoma cells, and it is a combination of multiple genes and mechanisms.

## Introduction

As everyone knows, hepatocellular carcinoma (HCC) is characterized by rapid growth and high malignancy (Li et al., 2019). Just a few patients can have surgery at early stages (Ma et al., 2017), and the postoperative recurrence rate of HCC is approximately as high as 50% (Bruix et al., 2015). This shows that the exploration of efficient and safe methods for HCC therapy is necessary. The combination of low-intensity and low-frequency ultrasound (US) and the sensitizers specifically in tumor tissue to produce cell cytotosis is the principle of sonodynamic therapy (SDT) (Trendowski, 2015). This method is an extension of photodynamic therapy (PDT). In recent years, the development of new sensitizers and the combination with PDT and SDT have been a hot research topic (Guo et al., 2020; Hao et al., 2020; Yao et al., 2020). However, PDT has some limitations. First, the effect is not significant for deep tumors because of the low tissue-penetrating depth of light, such as HCC (Gnerucci et al., 2020; Poderys et al., 2020). Second, the patients, after the injection of a photosensitizer, must be shielded from sunlight to avoid possible phototoxicity (Qian et al., 2016). US is a safe and valid imaging modality with intense penetration of human tissue, thereby improving the penetration limitation of PDT (Wood and Sehgal, 2015). In addition, SDT has some advantages, such as low toxicity, noninvasiveness, and excellent repeatability (Hu et al., 2015). The cavitation effect of US stimulates the sonosensitizers; thus, highly toxic reactive oxygen species (ROS) are produced to kill the tumor cells (Zhu et al., 2018; Zheng et al., 2020). The effect of the sonosensitizer in conjunction with the US promotes the apoptosis of tumor cells. SDT is effective in treating a variety of tumors (Um et al., 2021). Recently, the application of SDT has been studied extensively (Wang et al., 2021), and our team also showed that SDT could promote cell apoptosis through the mitochondrial pathway in HCC cells (Yang et al., 2021).

Hematoporphyrin monomethyl ether (HMME) has higher tumor selectivity than other porphyrin-related agents used as the sonosensitizer. When combined with US irradiation, it could have a pronounced cytotoxic effect (Jia et al., 2016), so it is widely used in the clinic (Ao et al., 2010). However, the light stability and water solubility of HMME are poor, so the body absorption is limited (Huang et al., 2018). Lonidamine (LND) was introduced in 1979; its essence is an indazole derivative (Huang et al., 2020) and is found to have antitumor activity by acting on tumor mitochondria. First, the proton-linked monocarboxylate transporter and mitochondrial pyruvate carrier are inhibited by LND (Nath et al., 2016); thus, the lactate emission and the uptake of pyruvate are influenced. Second, complexes I and II of the mitochondrial electron transport chain are distracted (Nath et al., 2016). Third, LND affects the mitochondrial permeability transition pore, disrupting mitochondrial transmembrane potential (Ravagnan et al., 1999). However, LND is mildly effective in inhibiting tumor development as a single chemotherapeutic agent (Huang et al., 2020). Recently, the combination of LND and chemotherapeutic agents or physical therapies has been researched; the results show that the anticancer effects of drugs or the therapeutic efficacy of physical therapies could be enhanced by the combination (Wu et al., 2019). In addition, the nanometer system encapsulates with LND to improve tumor targeting (Cheng et al., 2019). In the preliminary experiment, we prepared the target nanobubbles (NBs) conjugated with siRNA and confirmed its treatment effect to HCC *in vivo* and *in vitro* (Shang et al., 2019; Wu et al., 2021a).

Hence, novel NBs loaded with HMME and LND were structured in our study, which serves as the vehicle for the sonosensitizers and drugs. The introduction of NBs could improve the biocompatibility of HMME. The novel NBs achieve when combined with low-frequency US (LFUS) irradiation to the controlled release of HMME and LND, facilitating the efficacy of SDT. Furthermore, RNA sequencing was applied to investigate the mechanisms of SDT in our study. The whole transcriptome was investigated with next-generation high-throughput RNA-Seq, a high-throughput and quantitative technology (Ravagnan et al., 1999; Wu et al., 2019). The levels of transcripts and their isoforms could be presented more accurately (Shao et al., 2019; Chen et al., 2020). Thus, a new type of therapeutic approach and new gene targets can be found in HCC.

## Materials and Methods

### Preparation and Characterization of HMME-LND@C_3_F_8_-NBs

DSPC, DSPE-PEG-2000, and DSPE-PEG2000-biotin (Avanti Polar Lipids, Alabaster, AL) were collected to be liposomes at the ratio of 9:.5:.5 mg. Next, 10 mg of lipid powder, HMME and LND solution (at the concentration ratio of 2:1) was mixed in chloroform. We evaporated the mixture on the rotary evaporator at 45°C–50°C until the dry mixed lipid thin film on the bottom of the bottle was formulated. Then, the dry lipid film was dissolved in 10 ml phosphate-buffered saline (PBS) and vortexed. The mini-extruders (Avanti Polar Lipids, Alabaster, AL, United States) were used to prepare nano-level mixture lipid NBs. The HMME-LND mixed lipid solution was transferred into a sealed bottle in which air was replaced with C_3_F_8_ (Research Institute of Physical and Chemical Engineering of Nuclear Industry, Tianjin, China). After oscillation, the HMME-LND vesicles (HMME-LND@C_3_F_8_-NBs) were ready for characterization. HMME @C_3_F_8_-NBs were prepared in the same method, and the only difference is the absence of LND in the complex.

Scanning electron microscopy (SEM, Hitachi SU5000, Japan) and transmission electron microscopy (TEM, Hitachi TEM system, Japan) were applied to observe the HMME-LND@C_3_F_8_-NBs after fabrication. Dynamic light scattering (DLS, Zetasizer Nano ZS90, Malvern Instruments, United Kingdom) measured the size distribution and the zeta potential. Absorption spectra were recorded by the let–visible spectrophotometer (UV-Visible Spectrophotometer, Thermo Evolution 201, America).

### Cell Culture

The Institute of Cancer Research affiliated with the Harbin Medical University provided the HCC cell lines (Huh7 and HepG2), which the Ethics Committee approved. Dulbecco’s Modified Eagle Medium (DMEM, Hyclone, Logan, UT, United States) added 12% fetal bovine serum (FBS, Gibco, Carlsbad, CA) to cultivate HepG2 and Huh7 cells. The cells were placed in the incubator with 5% CO_2_ at 37°C.

### Cytotoxicity Assay With CCK-8

For the assay, 1 × 10^4^ cells of HepG2 and Huh7 were seeded into 96-well plates incubated for 24 h separately. Afterward, the fresh DMEM mixed with different concentrations of LND, HMME, HMME@C_3_F_8_-NBs, and HMME-LND@C_3_F_8_-NBs replaced the culture medium. US (1 MHz, 3.5 W/cm^2^) irradiated the cells incubated with HMME, HMME@C_3_F_8_-NBs, and HMME-LND@C_3_F_8_-NBs for 30 s. The cell counting kit 8 (CCK–8) assessed the cell viability of HepG2 and Huh7 after 24 h. The microplate reader (Promega Corp, Madison, WI, United States) detected the absorption of the plates at 450 nm wavelength. The combination index (CI) values were calculated using CompuSyn software19.

### Intracellular ROS Generation Detection and Mitochondrial Membrane Potential Assay

After the treatment with different groups combined with US (1 MHz, 3.5 W/cm^2^), HepG2 cells and Huh7 cells were incubated with DCFH–DA (Applygen Technologies Inc., Beijing, PR China) for 20 min, the concentration of DCFH–DA was 10 mmol/L.

The mitochondrial membrane potential for early apoptosis was detected with a JC-1 fluorescence probe (Beyotime, Jiangsu, China). First, JC-1 liquid was added in HepG2 and Huh7 cells for 20 min without light, and then, JC-1 staining buffer was used to wash the cells twice before taking photos with the fluorescence microscope. ImageJ software (National Institutes of Health, Bethesda, MD, United States) calculated the average intensity of fluorescence.

### Cell Apoptosis Assay

The annexin V-FITC Apoptosis Detection Kit (Beyotime, Jiangsu, China) tested the apoptosis of HepG2 and Huh7 cells; 8 × 10^4^ cells, which were washed with cold PBS, were counted and resuspended in 195 μl combination liquid, and then, those cells were stained with 5 μl Annexin V-FITC and 10 μl propidium iodide (PI). The apoptosis rates were measured with flow cytometry (BD Biosciences, United States) immediately. Annexin V-FITC+/PI− confirmed the apoptotic cells, and Annexin V-FITC+/PI + stained the necrotic cells.

### Measurement of Whole Transcriptome Library

The total RNAs before (HepG2 cells named Group A and Huh7 cells named Group C) and after (HepG2 cells named Group B and Huh7 cells named Group D) treated with HMME-LND@C_3_F_8_-NBs were isolated and tested. The following sequencing of genes was implemented by Novogene Bioinformatics Technology Cooperation (Beijing, China).

#### RNA-Seq of Samples

The raw material for the RNA-Seq was approximately 10 μg RNA per group. The index-coded samples were clustered with cBot Cluster Generation System (TruSeq PE Cluster Kit v3-cBot-HS, Illumia). Then, the libraries were sequenced on the Illumina HiSeq 2,500 platform, and the data were uploaded into NCBI’s Gene Expression Omnibus (https://www.ncbi.nlm.nih.gov/geo/query/acc.cgi?acc = GSE171857).

#### Data Analysis of Sequencing

In processing raw data, clean data with high quality for the following analyses were selected and calculated by eliminating ploy-N or low-quality reads.

#### Quantification of the RNA Expression Levels

Cuffdiff (v2.1.1) was applied to calculate the fragments per kilo-base of exon per million fragments mapped (FPKMs) of the RNAs (Trapnell et al., 2010), which was mainly based on the length of the fragments and the read count mapped to the fragment; it means fragments per kilo-base of exon per million fragments mapped (Wu et al., 2020).

#### Analysis of the Differential Genes

The adjusted *p*-value of differentially expressed genes was selected as <.05. The statistical routines provided from Cuffdiff were based on the negative binomial to determine differential expression in gene expression data (Trapnell et al., 2010).

The GO seqR package implemented the Gene Ontology (GO) enrichment analysis of differentially expressed RNAs (Young et al., 2010). The high-level functions and utilities of the biological system from the molecular level were explored by the Kyoto Encyclopedia of Genes and Genomes (KEGG) (Kanehisa et al., 2008). GO-seq and KOBAS software were applied in this step, and the corrected *p* < .01 was significantly enriched.

### Validation of RNA-Seq and Selection of lncRNAs

We selected some differentially expressed lncRNAs to reveal further clarifying mechanisms in the treatment of HCC on the gene level because of the complexity of lncRNAs. The differentially expressed genes between the two parallel experimental groups (FDR < .01, the FC value was in the same direction, and the absolute value was more significant than two) were screened to verify the accuracy of RNA-seq. Next, we compared the differential lncRNAs with the Lnc2Cancer (Gao et al., 2021) and LncRNADisease (Bao et al., 2019) database, The lncRNAs consistent with RNA-seq and associated with HCC from both databases were selected. Finally, the selected lncRNAs were validated with a molecular biology experiment.

### Quantitative Reverse Transcription-Polymerase Chain Reaction (qRT-PCR)

Trizol reagent (Thermo Fisher Scientific, Carlsbad, United States) was used to obtain the RNAs from HepG2 and Huh7 cells. Then, the researcher reverse transcribed the RNA to cDNA (Transcriptor First Strand cDNA Synthesis Kit, Roche, Switzerland, Germany). The qRT-PCR was performed by the SYBR Green real-time detection kit (CWBio, Beijing, China) with the CFX96 Detection System (Bio-Rad, California, United States). The relative expression of LINC00601 and ZFAS1 was calculated using the 2-ΔΔCt method and repeated three times. The following primers were used: LINC00601 (Forward: 5′-GAG​CTG​CAC​TGA​CCA​GTA​GG-3′, Reverse: 5′GTG​CTG​GCA​GAT​GGA​TCA​CT-3′); ZFAS1: (Forward: 5′-ACG​TGC​AGA​CAT​CTA​CAA​CCT-3′, Reverse: 5′-TAC​TTC​CAA​CAC​CCG​CAT-3′); GAPDH (Forward: 5′-CAT​GAG​AAG​TAT​GAC​AAC​AGC​CT-3′, Reverse: 5′-AGT​CCT​TCC​ACG​ATA​CCA​AAG​T-3′).

### Statistical Analysis

GraphPad Prism 8 software and Origin 2019 software performed statistical analysis. The experiments of HCC cells were repeated three times. The data were presented as an average of three replicates ±standard deviation; the *p* < .05 was considered statistically significant between control and samples values.

## Results

### Characterization of HMME-LND@C_3_F_8_-NBs

**FIGURE 1:**
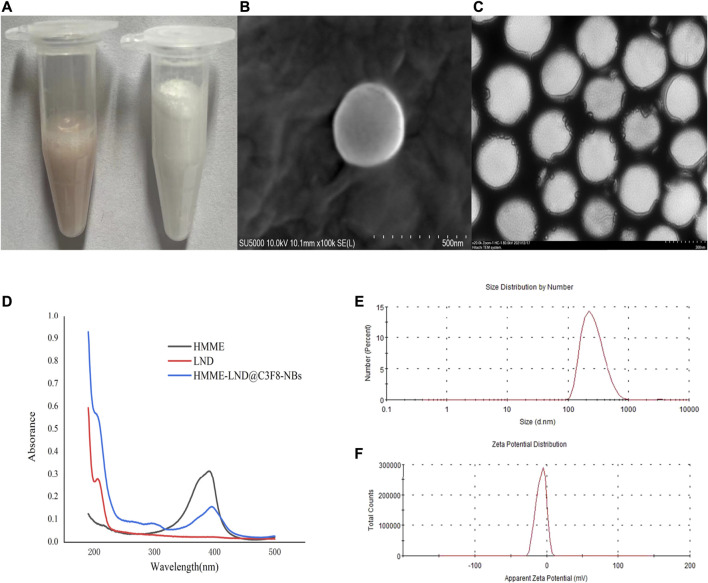
Fabrication and characterization of HMME-LND@C_3_F_8_-NBs. **(A)** The appearance of HMME-LND@C_3_F_8_-NBs (left) and pure NBs (right). HMME-LND@C_3_F_8_-NBs present with uniformly pink in color. **(B)** SEM image of HMME-LND@C_3_F_8_-NBs. **(C)** TEM image of HMME-LND@C_3_F_8_-NBs. **(D)** Absorption spectra of HMME, LND and HMME-LND@C_3_F_8_-NBs. **(E)** Size distribution of HMME-LND@C_3_F_8_-NBs by number. **(F)** Zeta potential distribution of HMME-LND@C_3_F_8_-NBs.

The appearance of pure NBs was shown as a uniform white emulsion (right of Figure 1A), and the HMME-LND@C_3_F_8_-NBs were rendered as a uniform pink emulsion (left of Figure 1A). Figures 1B,C displayed that HMME-LND@C_3_F_8_-NBs are shown as uniformly round under SEM and TEM. The size of the NBs was 284.76 ± 83.7 nm (Figure 1E). The zeta potential value of the complexes was –7.78 ± 6.72 mV (Figure 1F), and this showed that the HMME-LND@C_3_F_8_-NBs complexes were negatively charged. The absorbance peaks of HMME and LND were observed for the HMME-LND@C_3_F_8_-NBs (Figure 1D), indicating the joint of HMME and LND into NBs.

### Cytotoxicity Assay of HCC Cells

**FIGURE 2:**
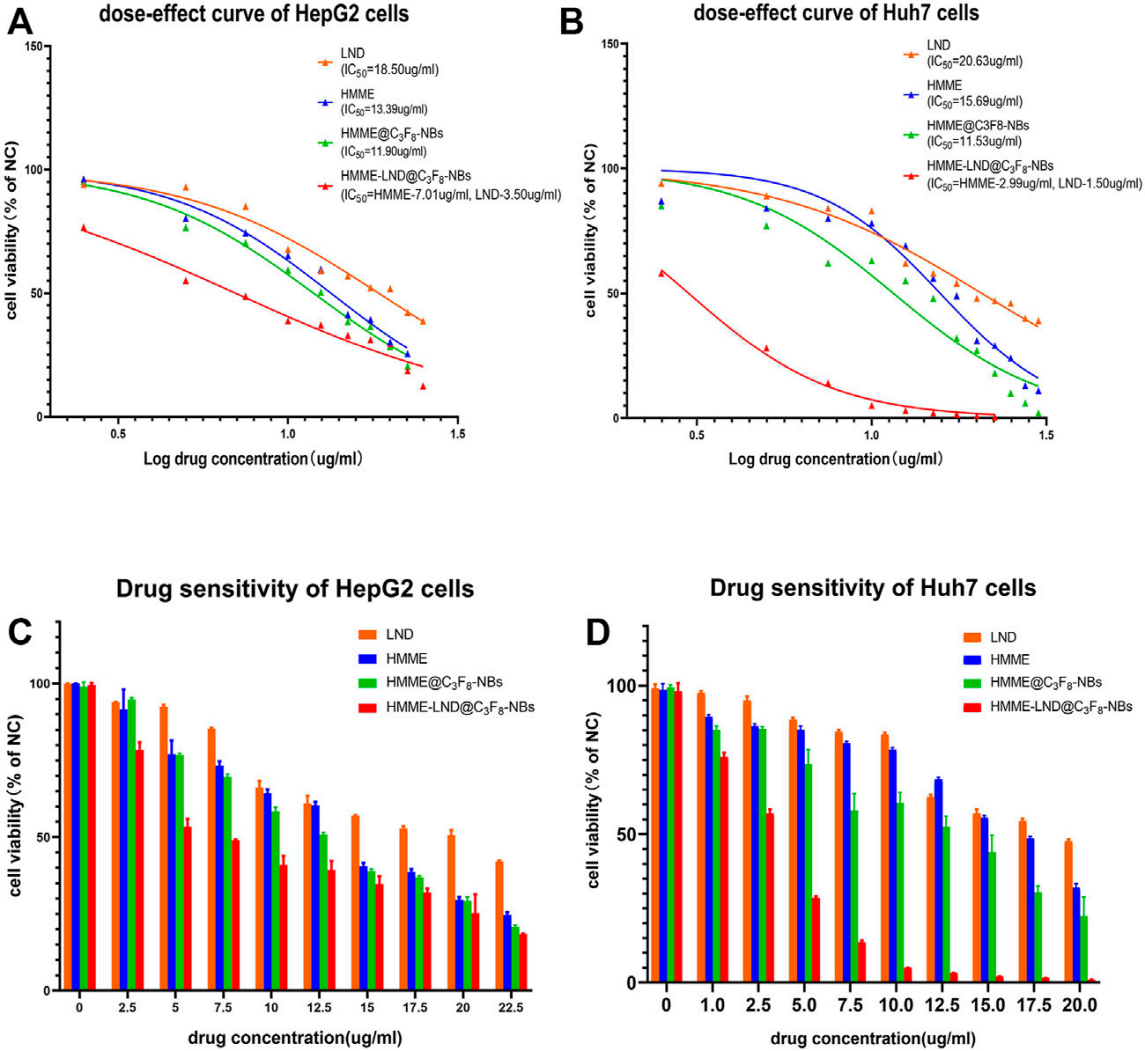
Comparison of drug resistance in the groups of LND, HMME, HMME @C_3_F_8_-NBs, and HMME-LND@C_3_F_8_-NBs. **(A)** The dose-effect curves of HepG2 cells to LND (orange curve), HMME (blue curve), HMME@C_3_F_8_-NBs (green curve), and HMME-LND@C_3_F_8_-NBs (red curve). **(B)** The dose-effect curves of Huh7 cells to LND (orange curve), HMME (blue curve), HMME@C_3_F_8_-NBs (green curve), and HMME-LND@C_3_F_8_-NBs (red curve). **(C)** Drug sensitivity of HepG2 cells to LND (orange column), HMME (blue column), HMME@C_3_F_8_-NBs (green column), and HMME-LND@C_3_F_8_-NBs (red column). **(D)** Drug sensitivity of Huh7 cells to LND (orange column), HMME (blue column), HMME@C_3_F_8_-NBs (green column), and HMME-LND@C_3_F_8_-NBs (red column).

The viability of HepG2, Huh7 cells was examined after interference with LND, HMME, HMME @C_3_F_8_-NBs, and HMME-LND@C_3_F_8_-NBs for 24 h. The results show that all the groups reduced the cell viability in a dose-dependent manner (Figures 2A,B, all *p* < .05). Figures 2A,B also show the half-maximal inhibitory concentration (IC50) of the four groups. The IC50 (shown as HMME/LND) of HMME-LND@C_3_F_8_-NBs in HepG2 cells and Huh7 cells were 7.01/3.50 μg/ml (CI value: .713) and 2.99/1.50 μg/ml (CI value: .264). The groups of HepG2 cells (Figure 2C) and Huh7 cells (Figure 2D) treated with HMME-LND@C_3_F_8_-NBs show more significant cytotoxicity than the other groups (all *p* < .05); the results reveal that NBs combined with HMME and LND decreased cell viability more significantly than LND alone, HMME alone, and NBs combined with HMME.

### Intracellular ROS Generation

**FIGURE 3:**
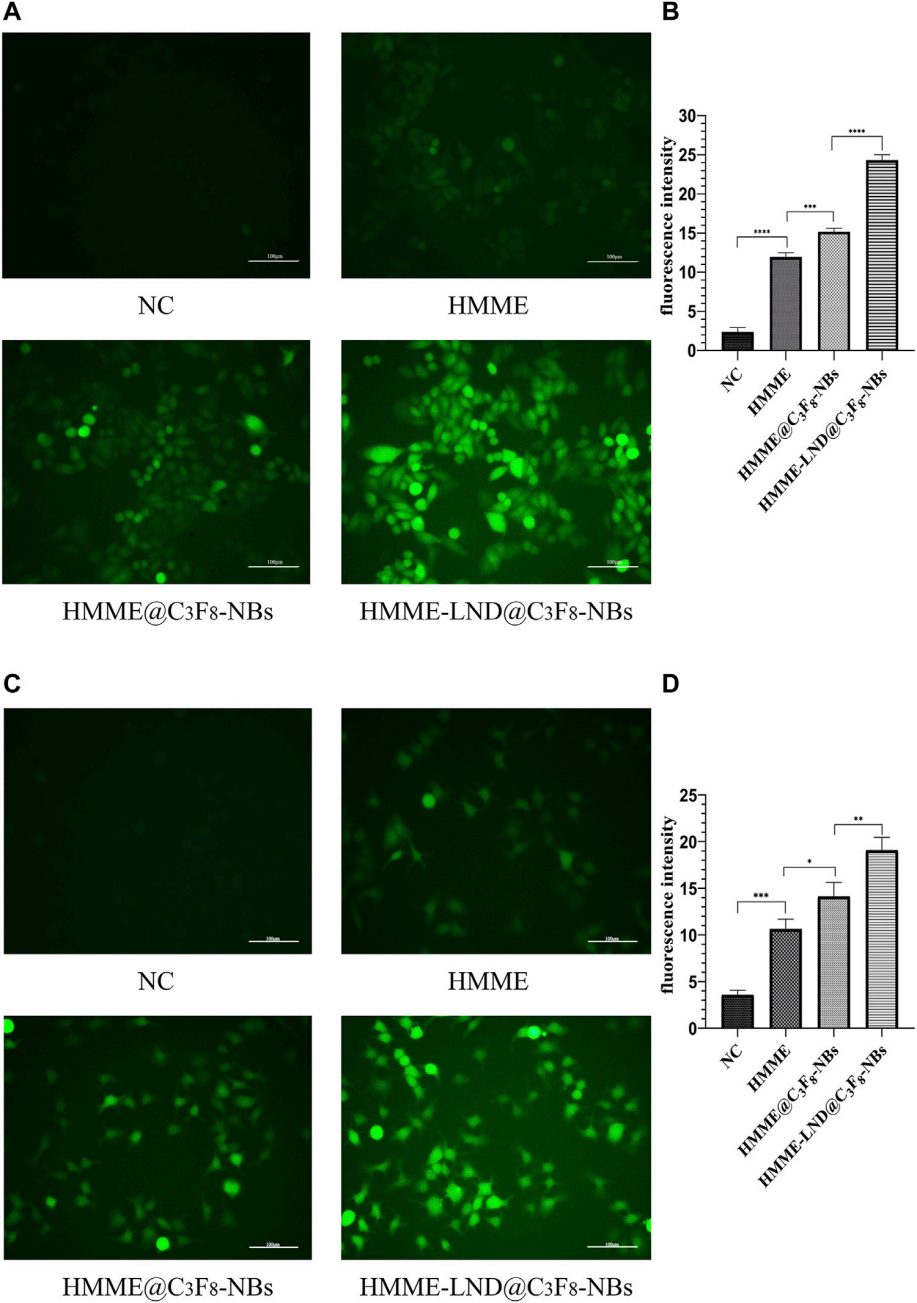
Image of intracellular ROS generation. **(A)** Fluorescence imaging of HepG2 cells stained by DCFH-DA. **(B)** The quantification of fluorescence intensity in four treatment groups of HepG2 cells. **(C)** Fluorescence imaging of Huh7 cells stained by DCFH-DA. **(D)** The quantification of fluorescence intensity in four treatment groups of Huh7 cells. *p < .05, **p < .01, ***p < .001, ****p < .0001.

The HMME-LND@C_3_F_8_-NBs groups in HepG2 and Huh7 cells exhibited more green fluorescence than the other three groups as shown in Figures 3A,C, which confirms that the HMME-LND@C_3_F_8_-NBs could be a more effective sonosensitizer generating ROS with LFUS. The fluorescence intensity of HMME @C_3_F_8_-NBs with LFUS was higher than HMME at the corresponding concentrations. Compared with the HMME @C_3_F_8_-NBs groups, LND improved the ability of the NBs complex to produce ROS. The quantification of fluorescence intensity in different groups of HepG2 and Huh7 cells was shown in Figures 3B,D. The fluorescence intensity of each group was statistically significant.

### Mitochondrial Membrane Potential Detection

**FIGURE 4:**
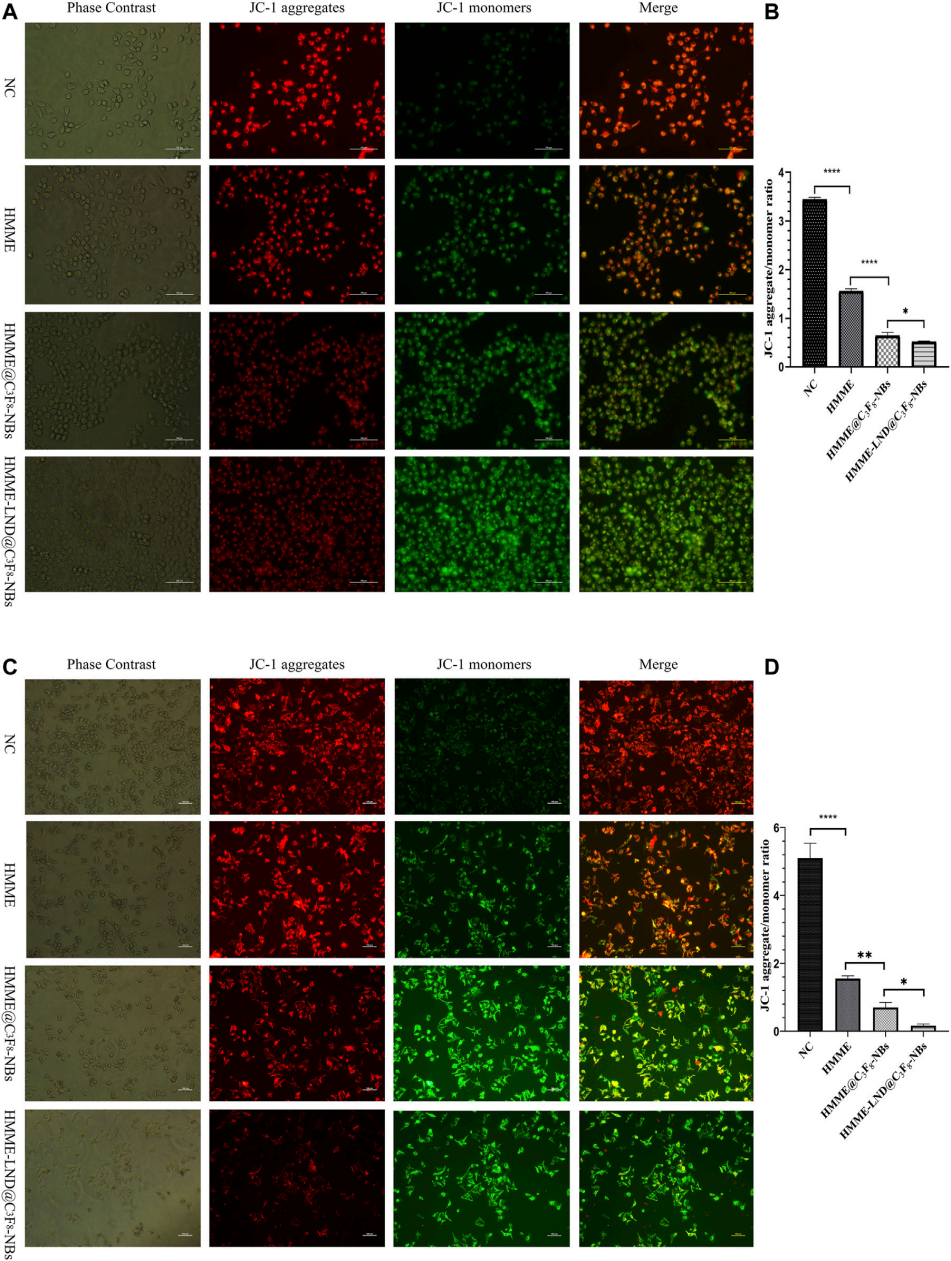
Mitochondrial membrane depolarization occurs during cell apoptosis. **(A)** Fluorescence imaging of HepG2 cells stained with JC-1 in different treatment groups. **(B)** The aggregate/monomer fluorescence intensity ratio of JC-1 in HepG2 cells. **(C)** Fluorescence imaging of Huh7 cells stained with JC-1 in different treatment groups. **(D)** The aggregate/monomer fluorescence intensity ratio of JC-1 in Huh7 cells. *p < .05, **p < .01, ***p < .001, ****p < .0001.

Fluorescence microscopic imaging was used to detect the mitochondrial membrane potential in HepG2 (Figure 4A) and Huh7 cells (Figure 4C). The mitochondrial membrane potential decreased after treatment with HMME-LND@C_3_F_8_-NBs more significantly than the control group, which showed conspicuous green fluorescence. NBs mediated with both HMME and LND were more evident than the NBs only mediated with HMME. The ratio of red to green fluorescence decreased memorably, quantifying fluorescence intensity as shown in Figures 4B,D. JC-1 aggregate converted to a monomeric form in the HepG2 cells, and Huh7 cells after SDT mediated with NBs complex, namely, early apoptosis occurred in the treatment group.

### The Cell Apoptosis of HCC Cells After SDT

**FIGURE 5:**
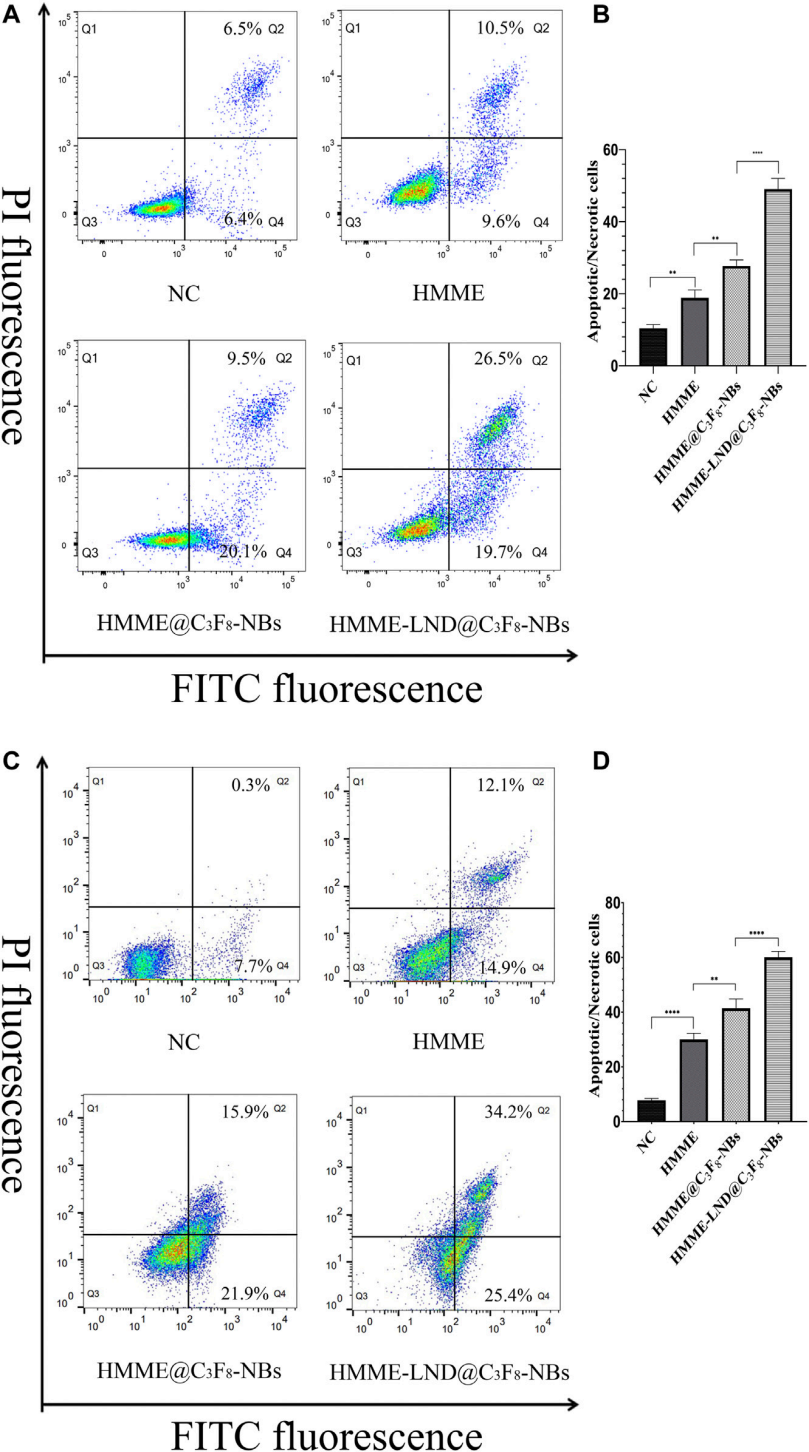
HMME-LND@C_3_F_8_-NBs induced cell apoptosis significantly. **(A)** HepG2 cells were treated with HMME, HMME@C_3_F_8_-NBs, and HMME-LND@C_3_F_8_-NBs for 24 h and then stained with Annexin-V and PI before being analyzed by flow cytometry. **(B)** The statistical results of the four groups of cell apop tosis in HepG2 cells. **(C)** Huh7 cells were treated with HMME, HMME@C_3_F_8_-NBs, and HMME-LND@C_3_F_8_-NBs for 24 h and then stained with Annexin-V and PI before being analyzed by flow cytometry. **(D)** The statistical results of the four groups of cell apoptosis in Huh7 cells. *p < .05, **p < .01, ***p < . 001, ****p < .0001.

HepG2 and Huh7 cells were stained with HMME, HMME @C_3_F_8_-NBs, and HMME-LND@C_3_F_8_-NBs combined with LFUS for 24 h. The results show that the proportion of apoptotic cells (Q4) and necrotic cells (Q2) in the HMME-LND@C_3_F_8_-NBs group was higher than the others (Figure 5). That is, the NBs mediated with HMME induced apoptosis in HepG2 and Huh7 cells, and the addition of LND amplified the effect significantly.

### RNA-Seq Analysis of mRNAs, lncRNAs, and circRNAs

**FIGURE 6:**
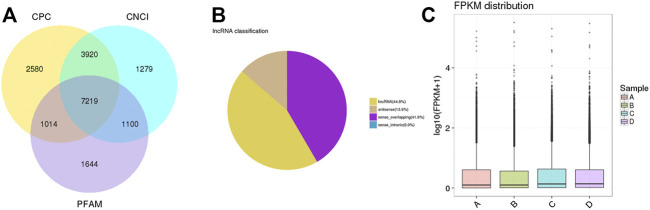
Coding potential analysis. **(A)** In this Venn diagram, three tools were selected to analyze the coding potentials of lncRNAs, including CNCI, CPC, and PFAM. A total of 7,219 lncRNA transcripts were filtered as novel lncRNAs. **(B)** The classification of filtered lncRNAs. **(C)** Boxplots showing the distribution of expression levels of genes.

Almost 361.8 million clean reads were isolated. The classification of raw reads is shown in Supplementary Figure S1. Almost 7,219 lncRNA transcripts were filtered as novel lncRNAs (Figure 6A). Those lncRNAs included 44.8% long intergenic noncoding RNAs (lincRNAs), 13.6% antisense lncRNAs, and 41.6% sense overlapping lncRNAs (Figure 6B). The distribution of expression levels of genes is shown with boxplots (Figure 6C).

### Analysis of Differential Expressed Genes

**FIGURE 7:**
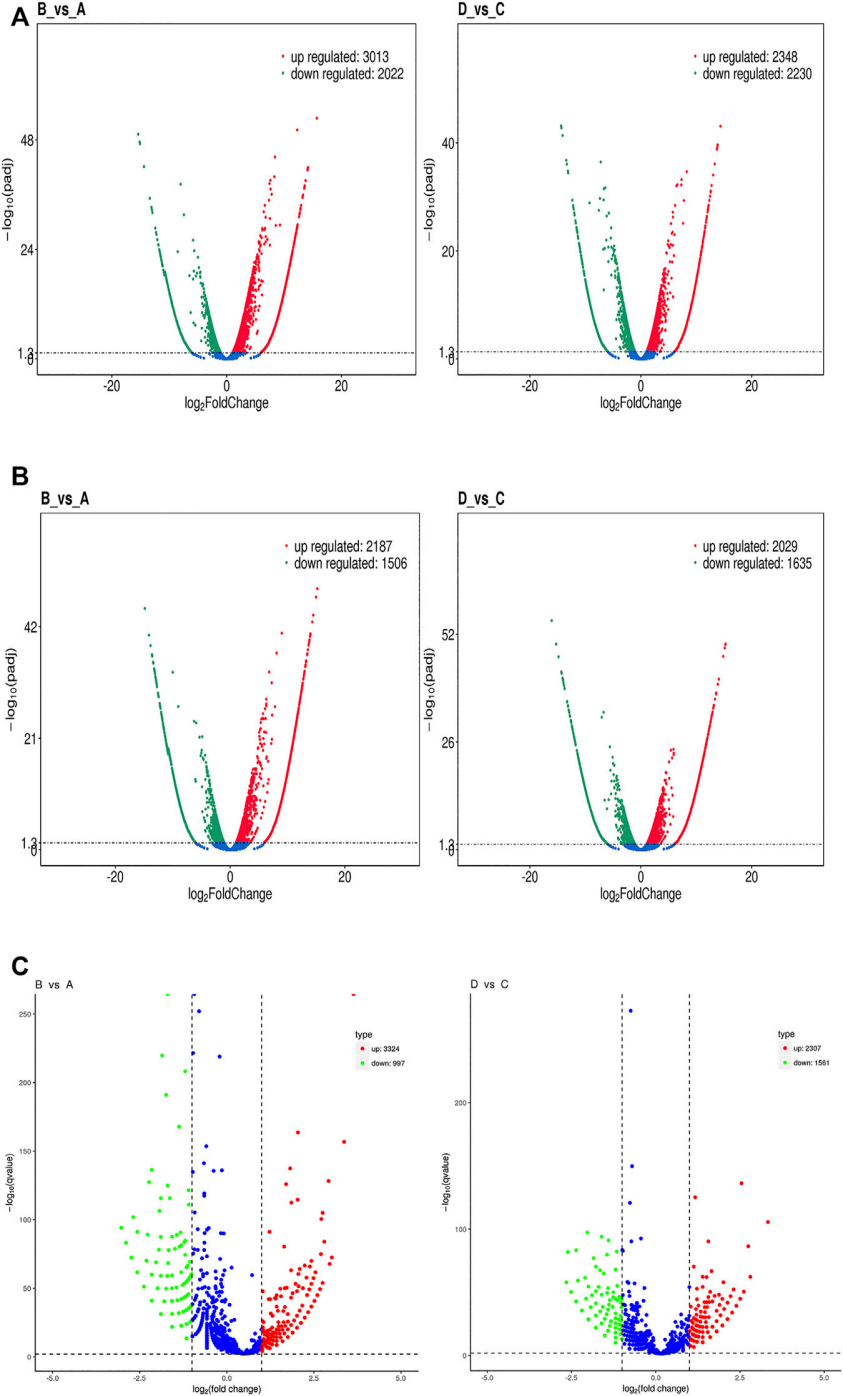
Volcano plots of RNA-Seq data distinguishing each experimental group. **(A)** A volcano plot of differentially expressed mRNA transcripts between groups B and A (left) and between groups D and C (right). **(B)** A volcano plot of differentially expressed lncRNA transcripts between groups B and A (left) and between groups D and C (right). **(C)** A volcano plot of differentially expressed circRNA transcripts between groups B and A (left) and between groups D and C (right). Each red dot denotes an individually upregulated transcript, and each green dot denotes an individually downregulated transcript.

**FIGURE 8:**
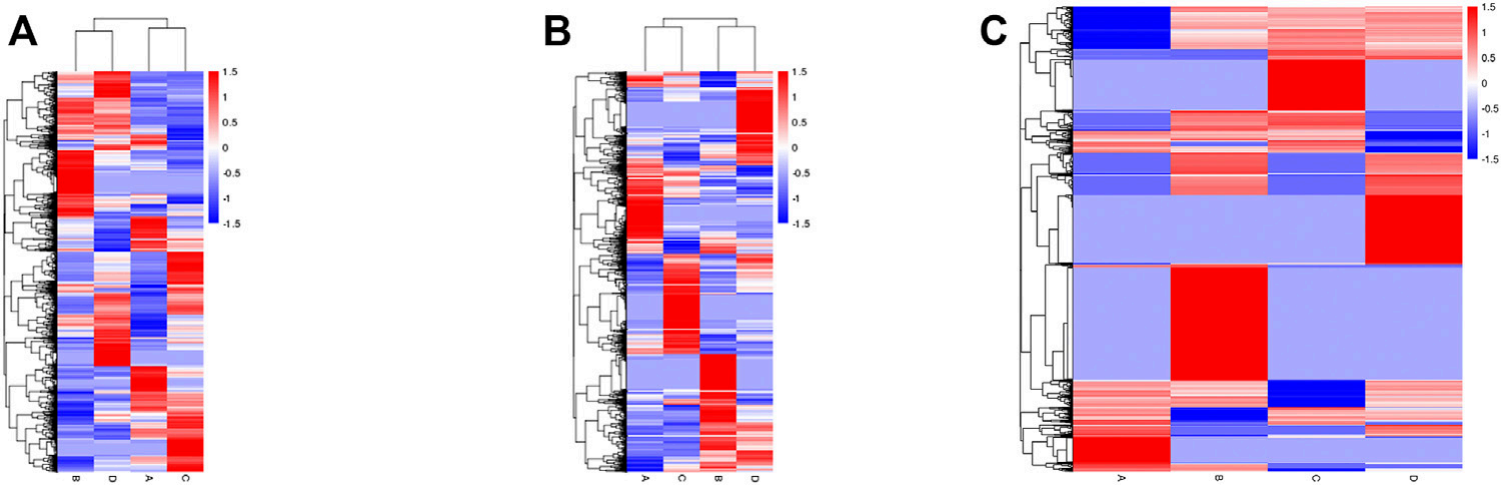
Heat map of hierarchical clustering. The expression profiles of differentially expressed mRNAs **(A)**, lncRNAs **(B)**, and circRNAs **(C)** of all experimental groups. Red indicates high and blue indicates low expression genes.

The level of mRNA, lncRNA, and circRNA were standardized based on the FPKM method. Differentially expressed mRNA, lncRNA, and circRNA were identified by edgeR software, the BH correction was performed on the obtained *p* values, and *p* < .05 were selected as differentially expressed mRNA, lncRNA, and circRNA. In total, 5,035 mRNA genes were differentially expressed between groups B and A, including 3,013 upregulated and 2022 downregulated genes (Figure 7A left); meanwhile, 4,578 mRNA genes were detected between groups D and C, including 2,348 upregulated and 2,230 downregulated transcripts (Figure 7A right). The expression of lncRNA in the groups also showed differences: 3,693 lncRNA (including 2,187 upregulated and 1506 downregulated genes) genes expressed differentially in group B vs. A (Figure 7B left); meanwhile, 3,664 lncRNA genes were found in group D vs. C, including 2029 upregulated and 1635 downregulated genes (Figure 7B right). Then, a total of 4,321 circRNA genes were expressed differentially between groups B and A, including 3,324 upregulated and 997 downregulated genes (Figure 7C left). Meanwhile, 3,868 circRNAs were differentially expressed between groups D and C, including 2,307 upregulated and 1561 downregulated genes (Figure 7C right). The above result of groups B/A and D/C were compared and shown with Venn pictures, 2,390 mRNA genes, 1556 lncRNA genes, and 1456 circRNA genes are the same part of the comparison result of the two samples (Supplementary Figure S2). In other words, those genes were differentially expressed in the two HCC cell lines after SDT; we call them stable differential genes of two parallel groups. The differentially expressed mRNAs, lncRNAs, and circRNAs were performed with cluster analysis, and the heat map (red and blue indicate high and low expression genes separately) shows the result according to log10 (FPKM+ 1) value (Figure 8). Groups A and B were similar to groups C and D separately, indicating that these genes were differentially expressed in HepG2 and Huh7 cells and could be used as biomarkers to predict their status.

### GO and KEGG Enrichment Analysis

**FIGURE 9:**
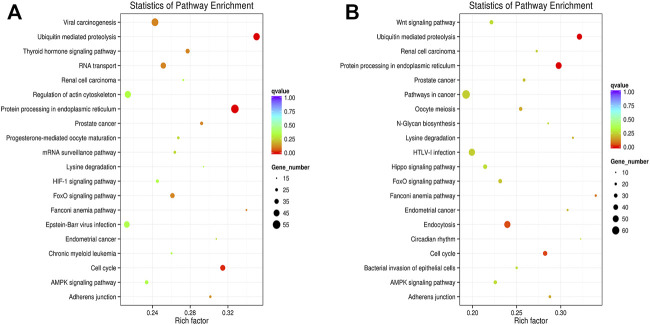
KEGG pathway analyses of dysregulated circRNA targets. **(A)** Analyses of top 20 over-represented KEGG pathways of dysregulated circRNAs between groups B and A. **(B)** Analyses of top 20 over-represented KEGG pathways of dysregulated circRNAs between groups D and C. The size of each dot stands as the number of significantly differentially expressed circRNA targets enriched in a corresponding pathway. The rich factor was calculated by dividing the number of enriched circRNA targets with the number of all background circRNA targets in a corresponding pathway.

In this analysis, 578 differential mRNA and 770 differential lncRNA target GO terms were enriched significantly between groups B and A (Supplementary Tables S1, S3, *p* < .01), whereas 638 mRNA GO and 693 lncRNA targets terms showed apparent enrichment between groups D and C (Supplementary Tables S2, S4, *p* < .01) based on the survey. In addition, 566 and 519 differentially expressed circRNAs GO terms enriched significantly in group B vs. A (Supplementary Table S5, *p* < .01) and group D vs. C (Supplementary Table S6, *p* < .01), respectively. The representative selected enriched terms of differential genes are shown in Tables 1–3. The detailed classification of GO terms of mRNA, lncRNA, and circRNA in different groups are shown in Supplementary Figure S3. A directed acyclic graph (DAG, Supplementary Figure S4) displays the hierarchical enrichment relations. Each node shows the name and the adjusted *p*-value of the terms. The NCBI’s Gene Expression Omnibus could be used to get the KEGG enrichment pathway. KEGG enrichment analysis of the differentially expressed mRNAs and lncRNA targets in the two parallel experimental groups did not simultaneously enrich into the same statistically significant pathway. Therefore, KEGG enrichment analysis of the stable differential genes in the two experimental groups was conducted. Some statistically significant pathways of mRNAs and lncRNA targets are listed in Supplementary Tables S7, S8, and the complete results are listed in Supplementary Tables S9, S10, respectively. Table 4 shows the top five pathway enrichments of differential circRNAs in the two groups. The results of pathway enrichments of circRNAs are shown in Figure 9.

**TABLE 1:** The representative results of GO enrichment analysis of the differential mRNAs in the two parallel experimental groups.

GO accession	Description	Term type	Adjusted *p* value^a^	Adjusted *p* value^b^
GO: 0006468	protein phosphorylation	biological_process	1.6263E-06	.00048562
GO:0001932	regulation of protein phosphorylation	biological_process	5.2165E-06	.00011407
GO:0045937	positive regulation of phosphate metabolic process	biological_process	.000012882	.00013544
GO:0015075	ion transmembrane transporter activity	molecular_function	3.7352E-08	1.8719E-12
GO:0034702	ion channel complex	cellular_component	1.6391E-06	2.9668E-09

a Means adjusted *p* value between Group B and Group A.

b Means adjusted *p* value between Group D and Group C.

**TABLE 2:** The representative results of GO enrichment analysis of the differential lncRNA targets in the two parallel experimental groups.

GO accession	Description	Term type	Adjusted *p* value^a^	Adjusted *p* value^b^
GO:0043167	ion binding	molecular_function	5.5825E-28	5.5048E-29
GO:0005739	mitochondrion	cellular_component	3.4134E-08	.029633
GO:0005524	ATP binding	molecular_function	1.2951E-07	.0015927
GO:0016310	phosphorylation	biological_process	1.4055E-06	1.0885E-08
GO:0001934	positive regulation of protein phosphorylation	biological_process	.0001431	2.0581E-06

a Means adjusted *p* value between Group B and Group A.

b Means adjusted *p* value between Group D and Group C.

**TABLE 3:** The representative results of GO enrichment analysis of the differential circRNA targets in the two parallel experimental groups.

GO accession	Description	Term type	Adjusted *p* value^a^	Adjusted *p* value^b^
GO:0000151	ubiquitin ligase complex	cellular_component	1.14E-11	3.04E-07
GO:0006915	apoptotic process	biological_process	5.14E-08	4.25E-07
GO:0016310	phosphorylation	biological_process	8.24E-08	4.94E-05
GO:0042981	regulation of apoptotic process	biological_process	.000516	2.31E-05
GO:0016462	Pyrophosphatase activity	molecular_function	3.45E-06	.0093758

a Means adjusted *p* value between Group B and Group A.

b Means adjusted *p* value between Group D and Group C.

**TABLE 4:** The representative results of KEGG enrichment analysis of the differential circRNA targets in the two parallel experimental groups.

Term	Database	ID	*p* value^a^	*p* value^b^
Ubiquitin mediated proteolysis	KEGG PATHWAY	hsa04120	1.84E-06	4.92E-06
Cell cycle	KEGG PATHWAY	hsa04110	.000112669	.00035239
FoxO signaling pathway	KEGG PATHWAY	hsa04068	.003657269	.00934791
AMPK signaling pathway	KEGG PATHWAY	hsa04152	.02464249	.016732793
Pathways in cancer	KEGG PATHWAY	hsa05200	.043844172	.012162988

a Means adjusted *p* value between Group B and Group A.

b Means adjusted *p* value between Group D and Group C.

### The Relative Expression of LINC00601 and ZFAS1

In the course of the current experiment, we intersected the differential lncRNAs screened in the two parallel experimental groups with the HCC-related genes that had been verified in the Lnc2Cancer and LncRNA Disease database, 12 meaningful lncRNAs were screened out, including seven upregulated lncRNAs and five downregulated lncRNAs (Table 5). LINC00601 and ZFAS1 were selected for validation in qRT-PCR due to their being expressed the same in the two databases and having significant differences in RNA-seq results.

**TABLE 5:** Differentially expressed lncRNAs after selection.

gene_name	B_FPKM	A_FPKM	log2FC.x	*p* value.x	padj.x	D_FPKM	C_FPKM	log2FC.y	*p* value.y	padj.y
MIR7-3HG	2.420705	.013773	7.287373499	2.46087E-23	.00	.579006	0	9.113273563	.00	.00
NEAT1	46.377953	6.550978	3.018352739	9.29666E-12	.00	29.989676	4.11039	3.023240091	.00	.00
DRAIC	.822032	.132606	3.262512013	2.07017E-11	.00	.477352	.079973	2.614969081	.00	.00
KRTAP5-AS1	.269793	.126184	2.116239064	.000414119	.00	2.025409	.27183	3.175907091	.00	.00
ZFAS1	394.174042	116.27299	2.095834651	9.19821E-07	.00	179.548859	76.2333	1.493727389	.00	.00
PURPL	.457486	.890473	1.552973405	.000233673	.00	.16079	.05791	1.841359637	.00	.00
LINC00346	7.25873	2.864223	1.641015005	.000104644	.00	1.896918	.7545	1.520242246	.00	.00
C1QTNF1-AS1	0	.686956	-11.13235733	1.30421E-22	.00	.269445	1.04175	-1.930826068	.00	.00
LINC00601	0	.166498	-9.298177857	5.03537E-13	.00	0	.03111	-6.757391023	.00	.00
FENDRR	1.23189	6.573173	-2.415616266	3.94492E-08	.00	.682777	1.526	-1.548923643	.00	.00
LINC01352	.014091	.181787	-3.302665951	.000102431	.00	0	.12009	-7.337901619	.00	.00
CASC15	.040926	.246925	-1.701054407	.000181455	.00	.069531	.51503	-2.316093931	.00	.00

A HepG2 cells; B HepG2 cells after SDT; C Huh7 cells; D Huh7 cells after SDT.

FPKM: the expression value of the genes in each group.

Log2FC:Log2FoldChange, multiples of differences between the two comparison groups.

Padj: the *p* value after correction.

**FIGURE 10:**
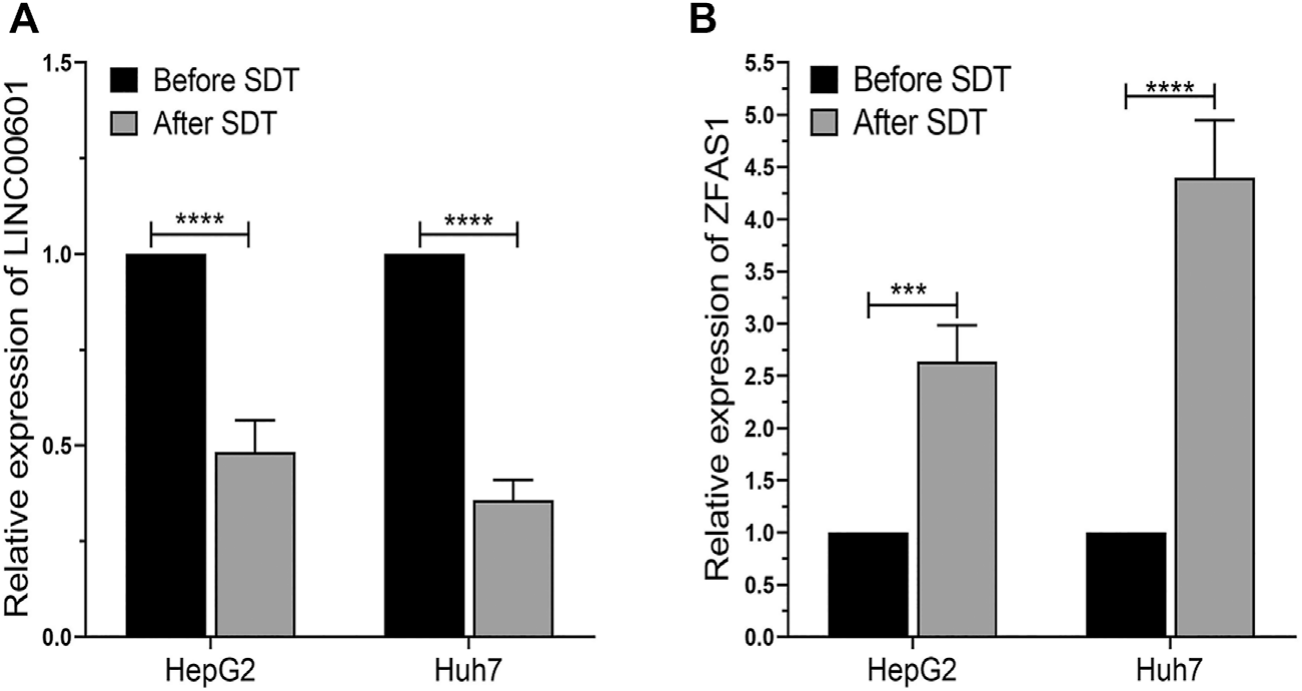
The qRT-PCR analysis of LINC00601 and ZFAS1 expression in HCC cells. **(A)** The downregulated expression of LINC00601 was observed in HCC cells after SDT with HMME-LND@C_3_F_8_-NBs. **(B)** The upregulated expression of ZFAS1 was observed in HCC cells after SDT with HMME-LND@C_3_F_8_-NBs. ***p < .001, ****p < .0001.

The expression of LINC00601 and ZFAS1 in HepG2 and Huh7 cells after SDT was measured through qRT-PCR. The results confirmed that the expression of LINC00601 was reduced in HepG2 and Huh7 cells after SDT. Meanwhile, the expression of ZFAS1 was upregulated after SDT in HCC cell lines (Figure 10). These results were consistent with RNA sequencing and related lncRNA databases, so we selected LINC00601 and ZFAS1 as representatives for further research.

## Discussion

HMME-LND@C_3_F_8_-NBs was structured in the study to improve the intractable problem of HMME, such as poor light stability. LND was added to the NBs as a sensitizer to enhance the efficacy of SDT. The gas core and the nanoscale diameter of HMME-LND@C_3_F_8_-NBs make the complexes able to be used as sonosensitizers of SDT and can be used to enhance the cavitation effects of NBs. We developed new sonosensitizer delivery to improve the effect of SDT. The synergy of LFUS irradiation and NB complexes mediated with HMME and LND achieved encouraging tumor cytotoxicity as shown in Figure 2. The previous study proved that sonosensitizer conjugated with NBs could enhance the effect of SDT (Ma et al., 2019). In the present study, we detected excessive ROS in the HepG2 and Huh7 cells treated with the HMME-LND@C_3_F_8_-NBs under LFUS. ROS production in the groups treated with simple HMME solution and HMME @C3F8-NBs was lower than the HMME-LND@C_3_F_8_-NBs groups significantly (Figure 3). These results confirm that HMME-LND@C_3_F_8_-NBs play an efficacious nanosonosensitizer during the SDT treatment of HCC.

Mitochondria plays an essential role in cell apoptosis; the decrease of mitochondrial membrane potential instructed the apoptosis in HCC cells (Kroemer et al., 2007). In the process of SDT, the stability of the mitochondrial membrane is damaged by ROS-induced oxidative injury. Mitochondria releases cytochrome C, which conjugates the apoptotic protein activator, inducing cell apoptosis (Li et al., 2016). It is confirmed that the effect of LND on tumor cells was also concentrated in tumor mitochondria, which may be why it can enhance the SDT effect. The JC-1 method was applied to investigate the damage of mitochondria after SDT. The treatment of HMME-LND@C_3_F_8_-NBs indicates mitochondrial dysfunction (Figure 4). Furthermore, the HMME-LND@C_3_F_8_-NBs groups obtained a more considerable degree of apoptosis for the HepG2 and Huh7 cells (Figure 5). As we all know, the migration and invasion of cancer cells are also related to the mitochondrial function, so the transwell assay was performed to investigate the migration and invasion ability of HCC cells after different treatment (Supplementary Figures S5, S6). The results show that NBs combined with HMME and LND suppressed the migration and invasion ability of HCC cells more than HMME alone and NBs combined with HMME, and the above groups were combined with US irradiation.

Therefore, we confirm that HMME-LND@C_3_F_8_-NBs combined with LFUS effectively promote cell apoptosis. However, the genes or pathways that related to SDT were unclear. RNA-seq is confirmed to achieve the purpose of understanding the genes and pathways after SDT (Wu et al., 2021b). The study’s objective was to explore the mRNAs, lncRNAs, and circRNAs that promoted cell apoptosis in HCC. According to our results, some particular genes might be considered potential targets for the proper treatment of HCC.

The differential expressions of mRNAs, lncRNAs, and circRNAs in HCC cells were identified after SDT, and similar expressions appeared in the 2 cell lines (Figure 7). Aggregately, 2,390 mRNAs, 1556 lncRNAs, and 1456 circRNAs significantly dysregulated were identified (Supplementary Figure S2). Those differentially expressed genes were predicted their roles in HCC preliminarily. GO found that the differential mRNAs were partly enriched in “protein phosphorylation,” “regulation of protein phosphorylation,” “positive regulation of phosphate metabolic process,” “ion transmembrane transporter activity,” and “ion channel complex,” and these enriched terms were related to phosphorylation or related content, indicating that phosphorylation might be a mechanism in apoptosis of HCC cells promoted by SDT. Also, some lncRNA target enrichment (Table 2) might regulate different molecular biological functions. As we can see, not only is the phosphorylation still involved in this progress, but also other biological processes were impacted, such as the mitochondrial function. This was consistent with our previous experimental results. In addition, five characterizing differential circRNA enrichment terms were selected in Table 3, and it involved many complex processes, such as apoptosis, phosphorylation, and ubiquitination. Therefore, apoptosis of HCC cells caused by SDT might result from synergistic affection between multiple biological processes.

The enriched pathways were studied to discover the mechanism of SDT in this study. The differentially expressed transcriptome genes were enriched into multiple pathways through KEGG enrichment analysis; some representative terms are listed in Table 4, Supplementary Tables S7, S8, including “transcriptional misregulation in cancer,” “viral carcinogenesis,” “ubiquitin mediated proteolysis,” “cell cycle,” “pathways in cancer,” and some classic pathways in tumorigenesis and development. It can be seen that the mechanism of apoptosis induced by SDT was complex, and it involved multiple pathways, such as ubiquitination and phosphorylation. Without a doubt, there was an explicit change between the whole transcriptome in HCC after SDT.

Recently, researchers have confirmed that lncRNAs are involved in multiple biological processes. A variety of specifically dysregulated lncRNAs are confirmed in many kinds of cancer, and those genes can be seen as the potential diagnostic or prognostic biomarkers (Shi et al., 2013). In our study, two types of HCC cells were treated with SDT using a novel compound sonosensitizer, and 1556 lncRNAs were shown dysregulated in HCC cells after treatment. Some simple bioinformatics methods selected two lncRNAs (LINC00601 and ZFAS1) for verification and study.

Only a few studies have been reported on the lncRNA-LINC00601, especially its impact on the occurrence and development of HCC. Y.C Wang’s research finds that LINC00601 is upregulated in HCC tissue, and si-LINC00601 might inhibit the proliferation and promote the apoptosis of HCC cells; the downregulation of LINC00601 can suppress the activation of the MAPK signaling pathway (Wang et al., 2020). Our study shows that the expression of LINC00601 decreased significantly after SDT; the apoptosis of HCC cells increased significantly. This conclusion is consistent with the previous research. In summary, we believe that LINC00601 could be an oncogenic gene in HCC, and it may be considered a new target for gene therapy of HCC in the future.

ZFAS1 (NFX1-type zinc finger-containing protein one anti-sense RNA 1) is another lncRNA screened in our research to be studied. ZFAS1 is related to multiple types of tumors, such as breast cancer (Askarian-Amiri et al., 2011), renal cell carcinoma (Dong et al., 2019), gastric cancer (Zhou et al., 2016), and colorectal cancer (Wang and Xing, 2016). However, its role is different in those tumors. ZFAS1 is upregulated in most tumor tissue, and it may promote the metastasis and development of tumors. ZFAS1 was significantly downregulated in breast cancer tissue so that it can be considered a tumor suppressor. Nevertheless, the role of ZFAS1 in HCC remains controversial. T. Li’s research suggests that ZFAS1 bonds miR-150 and abrogates its tumor-suppressive function to be an oncogene in HCC development (Li et al., 2015). Nevertheless, T. Wang reports that ZFAS1 regulates methylation of miR-9 and is a potential tumor suppressor in HCC (Wang et al., 2016). The latter is similar to our current research. ZFAS1 is found significantly upregulated after SDT with HMME-LND@C_3_F_8_-NBs. That is, the ZFAS1 may be a tumor suppressor factor in the development of HCC, but the specific mechanism of the process remains to be studied in the future.

However, there are still limitations in this study. First, the HMME-LND@C_3_F_8_-NBs complex obtained is not confirmed to be safe *in vivo*. Second, only two HCC cell lines were used for qRT-PCR and RNA-seq; moreover, the gene difference between HCC cells and normal hepatocytes was not analyzed, leading to biases. Third, our study focuses only on HMME as sonosensitizers, and it is unknown whether other types of sonosensitizers affect the SDT sensitivity or the gene difference on HCC.

## Conclusion

This study provides that the HMME-LND@C_3_F_8_-NBs complex can produce a mass of ROS and significantly reduce the mitochondrial membrane potential with LFUS, promoting HCC cells apoptosis effectively. Furthermore, the result shows various mRNAs, lncRNAs, and circRNAs for further research concerning their precise functions in HCC. LINC00601 and ZFAS1 may be used as new biomarkers for the treatment of HCC. This research provides a new type of sonosensitizer for the SDT of HCC and proposes a new clinical treatment strategy and research direction for HCC.

## Data Availability

The datasets presented in this study can be found in online repositories. The names of the repository/repositories and accession number(s) can be found in the article/Supplementary Material.

## Abbreviations

RNA-seq, RNA sequencing; SDT, sonodynamic therapy; LND, lonidamine; HMME, hematoporphyrin monomethyl ether; HCC, hepatocellular carcinoma; US, ultrasound; PDT, photodynamic therapy; ROS, reactive oxygen species; PT, permeability transition; NBs, nanobubbles; LIFU, low intensity focused ultrasound; PBS, phosphate-buffered saline; GO, gene ontology; KEGG, kyoto encyclopedia of genes and genomes; MMP, mitochondrial membrane potential.
