# Supplementary material for: RNA-Seq Technology Reveals the Mechanism of SDT Combined With Novel Nanobubbles Against HCC
Source: Front Mol Biosci. 2022 Feb 7;8:791331. doi: 10.3389/fmolb.2021.791331 (PMC8859324; doi:10.3389/fmolb.2021.791331)
Supplement: Supplementary file 4 [file Table8.DOCX]

TABLE S7 The representative results of KEGG enrichment analysis of stable differential mRNAs of two parallel experimental groups

| Term | Database | ID | *P* value |
| --- | --- | --- | --- |
| Transcriptional misregulation in cancer | KEGG PATHWAY | hsa05202 | 8.99E-04 |
| PI3K-Akt signaling pathway | KEGG PATHWAY | hsa04151 | 0.015221777 |
| Viral carcinogenesis | KEGG PATHWAY | Hsa05203 | 0.01572936 |
| cGMP-PKG signaling pathway | KEGG PATHWAY | hsa04022 | 0.016174016 |
| MAPK signaling pathway | KEGG PATHWAY | hsa04010 | 0.023827741 |
