# Supplementary figures and images for: RNA-Seq Technology Reveals the Mechanism of SDT Combined With Novel Nanobubbles Against HCC
Source: Front Mol Biosci. 2022 Feb 7;8:791331. doi: 10.3389/fmolb.2021.791331 (PMC8859324; doi:10.3389/fmolb.2021.791331)

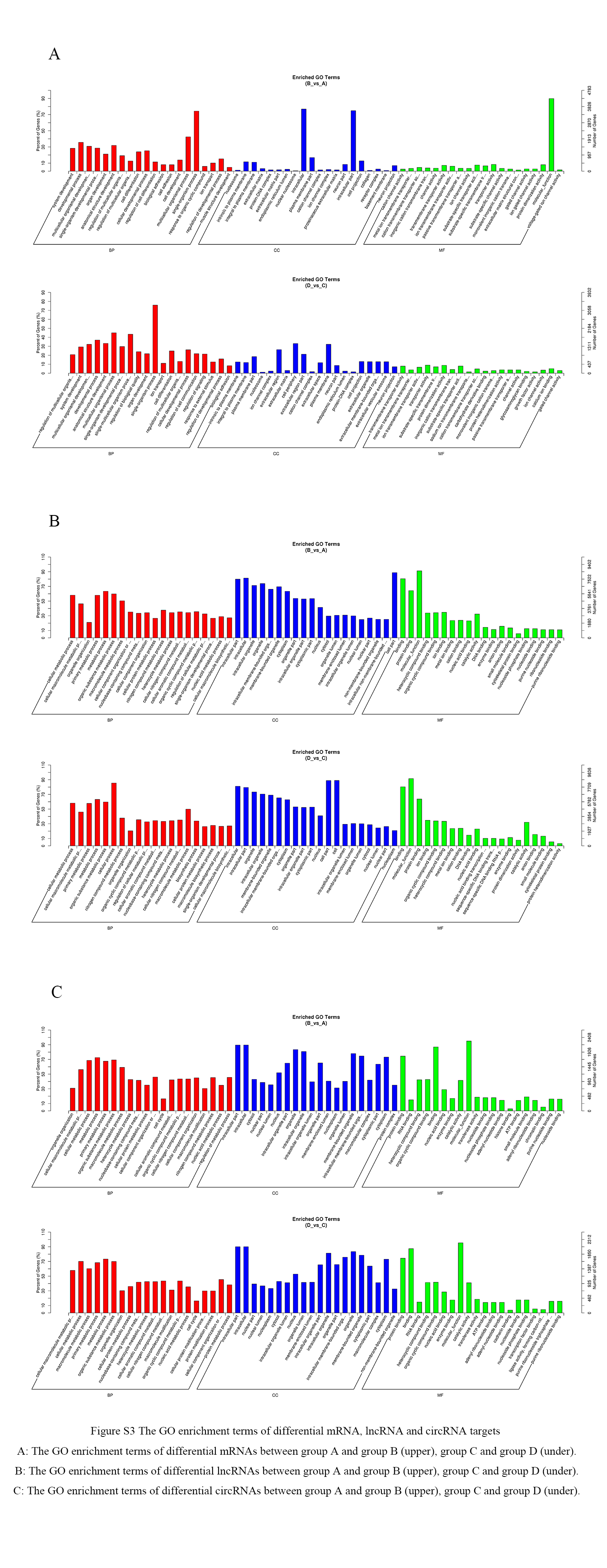

Supplement: Supplementary file 6 [file Image3.TIF]

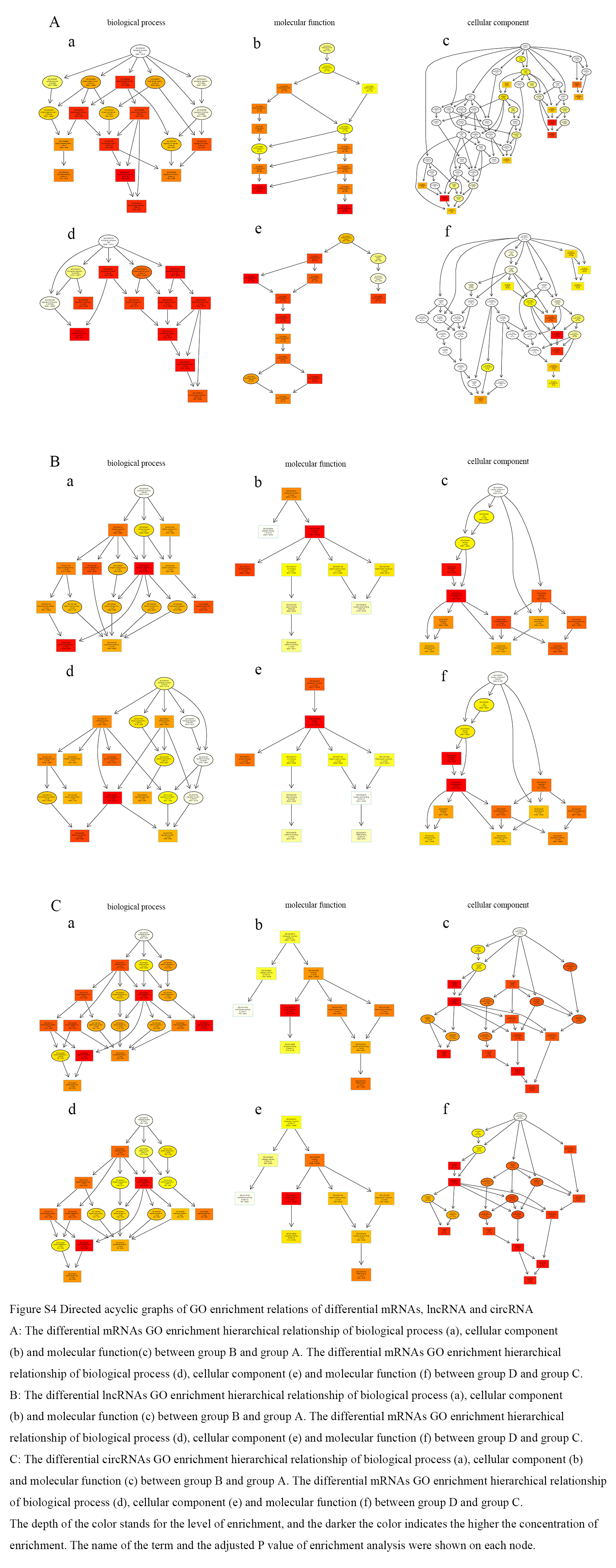

Supplement: Supplementary file 7 [file Image4.TIF]

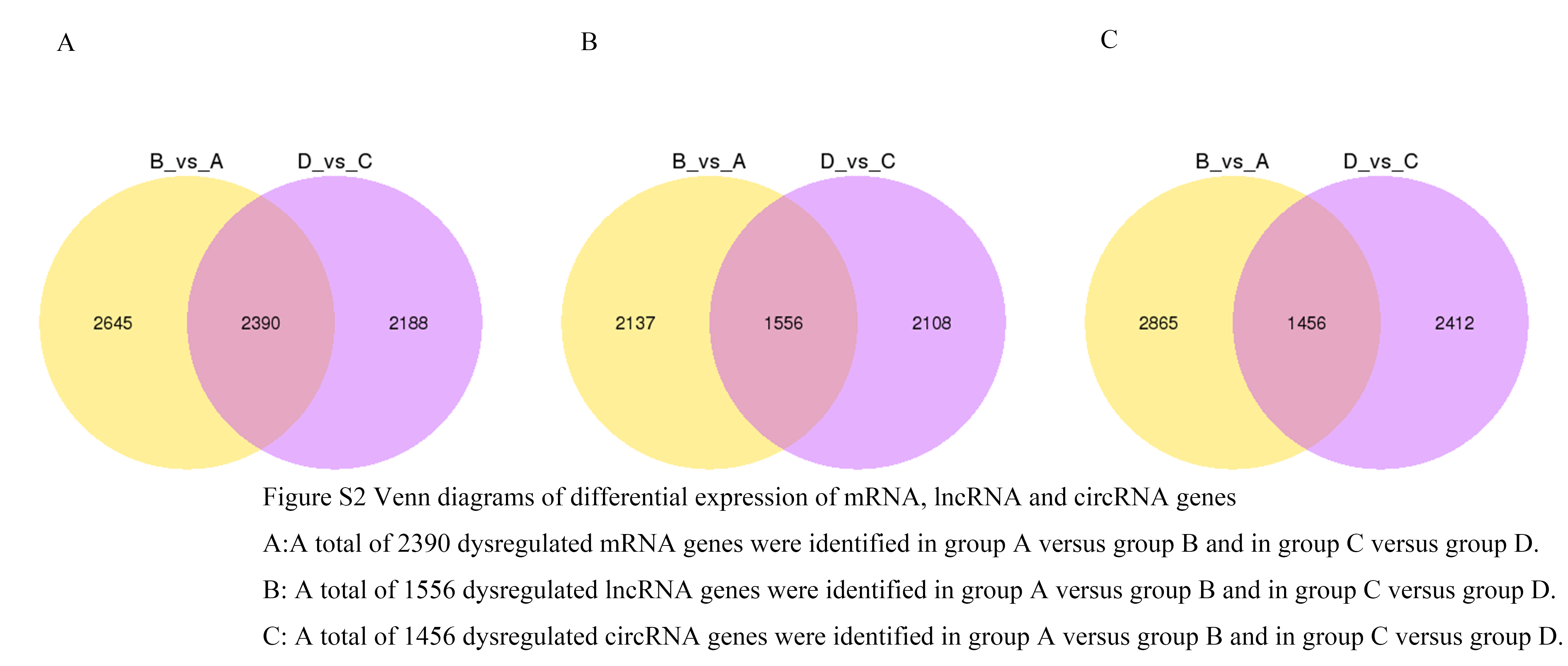

Supplement: Supplementary file 9 [file Image2.TIF]

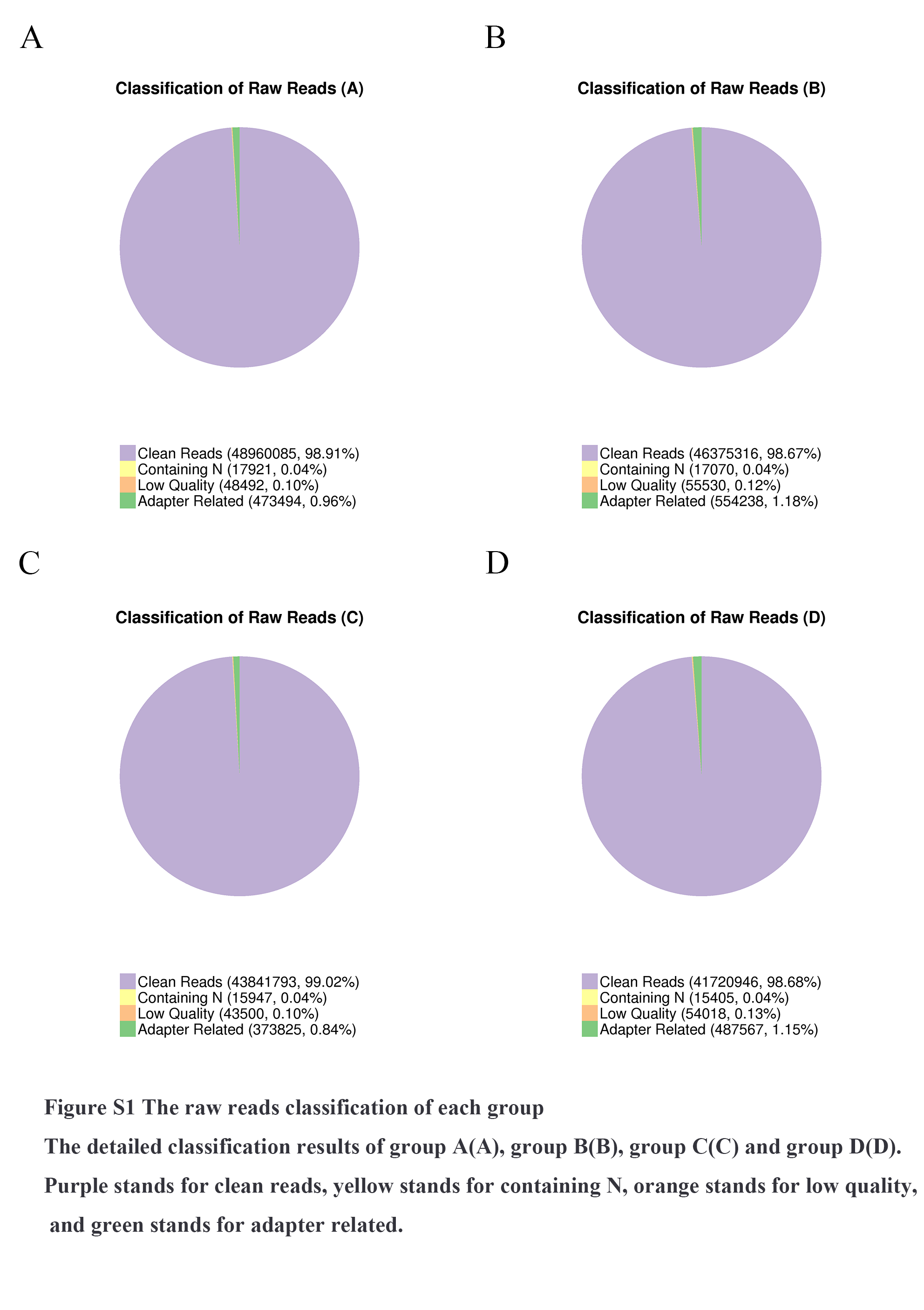

Supplement: Supplementary file 11 [file Image1.TIF]
