# Supplementary material for: RNA-Seq Technology Reveals the Mechanism of SDT Combined With Novel Nanobubbles Against HCC
Source: Front Mol Biosci. 2022 Feb 7;8:791331. doi: 10.3389/fmolb.2021.791331 (PMC8859324; doi:10.3389/fmolb.2021.791331)
Supplement: Supplementary file 14 [file Table11.DOCX]

TABLE S8 The representative results of KEGG enrichment analysis of stable differential lncRNA targets of two parallel experimental groups

| Term | Database | ID | *P* value |
| --- | --- | --- | --- |
| Transcriptional misregulation in cancer | KEGG PATHWAY | hsa05202 | 0.001633242 |
| MAPK signaling pathway | KEGG PATHWAY | hsa04010 | 0.002889091 |
| Viral carcinogenesis | KEGG PATHWAY | Hsa05203 | 0.003446921 |
| Ras signaling pathway | KEGG PATHWAY | hsa04014 | 0.019731073 |
| mTOR signaling pathway | KEGG PATHWAY | hsa04150 | 0.021647433 |
